# The protein PprI provides protection against radiation injury in human and mouse cells

**DOI:** 10.1038/srep26664

**Published:** 2016-05-25

**Authors:** Yi Shi, Wei Wu, Huiping Qiao, Ling Yue, Lili Ren, Shuyu Zhang, Wei Yang, Zhanshan Yang

**Affiliations:** 1 Department of Radiation Toxicology, School of Radiological Medicine and Protection, Medical College of Soochow University, Collaborative Innovation Center of Radiation Medicine of Jiangsu Higher Education Institutions, Soochow University, Suzhou, Jiangsu 215123, China; 2 Department of Radiation Genetics, School of Radiological Medicine and Protection, Medical College of Soochow University, Collaborative Innovation Center of Radiation Medicine of Jiangsu Higher Education Institutions, Soochow University, Suzhou, Jiangsu 215123, China; 3 Department of Radiobiology, School of Radiological Medicine and Protection, Medical College of Soochow University, Collaborative Innovation Center of Radiation Medicine of Jiangsu Higher Education Institutions, Soochow University, Suzhou, Jiangsu 215123, China

## Abstract

Severe acute radiation injuries are both very lethal and exceptionally difficult to treat. Though the radioresistant bacterium *D. radiodurans* was first characterized in 1956, genes and proteins key to its radioprotection have not yet to be applied in radiation injury therapy for humans. In this work, we express the *D. radiodurans* protein PprI in *Pichia pastoris* yeast cells transfected with the designed vector plasmid pHBM905A-*pprI*. We then treat human umbilical endothelial vein cells and BALB/c mouse cells with the yeast-derived PprI and elucidate the radioprotective effects the protein provides upon gamma irradiation. We see that PprI significantly increases the survival rate, antioxidant viability, and DNA-repair capacity in irradiated cells and decreases concomitant apoptosis rates and counts of damage-indicative γH2AX foci. Furthermore, we find that PprI reduces mortality and enhances bone marrow cell clone formation and white blood cell and platelet counts in irradiated mice. PprI also seems to alleviate pathological injuries to multiple organs and improve antioxidant viability in some tissues. Our results thus suggest that PprI has crucial radioprotective effects on irradiated human and mouse cells.

Industrial, agricultural, and medical applications of ionizing radiation have proven highly beneficial to humankind. However, high-doses of ionizing radiation incurred from nuclear accidents, tumor radiotherapy, spaceflight, and other sources can cause severe acute radiation injuries (ARIs). The successful treatment of severe ARIs presents an enormous challenge to medical professionals. Therapies for radiation injury and practices designed to prevent high radiation exposure are both active topics of research.

The bacterium *Deinococcus radiodurans* (*D. radiodurans*) is one of the most radioresistant life forms known to exist^1^,^2^. Its extraordinary resistance to both particulate and electromagnetic forms of ionizing radiation, driven by a high capacity for DNA repair, has attracted considerable interest among radiobiologists. The specific mechanisms by which *D. radiodurans* protects itself from radiation damage remain somewhat uncertain. Current research indicates that a tight, ring-like chromosomal structure and an efficient means for repairing DNA double-strand breaks (DSBs) are crucial elements of its radioresistance^1^,^3^,^4^.

The gene *pprI* (inducer of pleiotropic proteins promoting DNA repair; also called *irrE*) is known to play a central regulatory role in DNA repair that occurs within *D. radiodurans* after irradiation. Its expression product (an uncharacterized protein, PprI, generated by coding sequence DR0167) stimulates transcription and translation of RecA and other DNA repair genes in response to acute radiation damage^5^,^6^,^7^. Proteomics analyses suggest PprI serves as a general switch for upregulating 31 different proteins, two of which (RecA and PprA) are well known for their roles in ameliorating damage to DNA^8^. In *Escherichia coli*, exogenous expression of PprI both enhances the expression of RecA and improves catalase activity, factors that both promote DNA repair and protect against oxidative damage^7^. In addition, expression of *pprI* gene in yeast is known to enhance resistance to extreme environments and increase the yield of alcoholic fermentation^9^.

*D. radiodurans*, of course, is a prokaryotic organism. Differences in gene composition, modes of protein expression, and codon preferences between bacteria and highly diverged eukaryotes complicate the application of PprI to improving radioresistance in organisms like humans. Notably, PprI has no homologue in mammalian cells. In this work, we investigate whether the prokaryotic *pprI* gene can be efficiently expressed in the yeast species *Pichia pastoris* and, furthermore, whether the expression product can have any effects on radioresistance in irradiated human and mouse cells. To the best of our knowledge, no results from the literature presently address these subjects of inquiry.

## Results

### PprI is highly expressed in yeast

**Figure 1:**
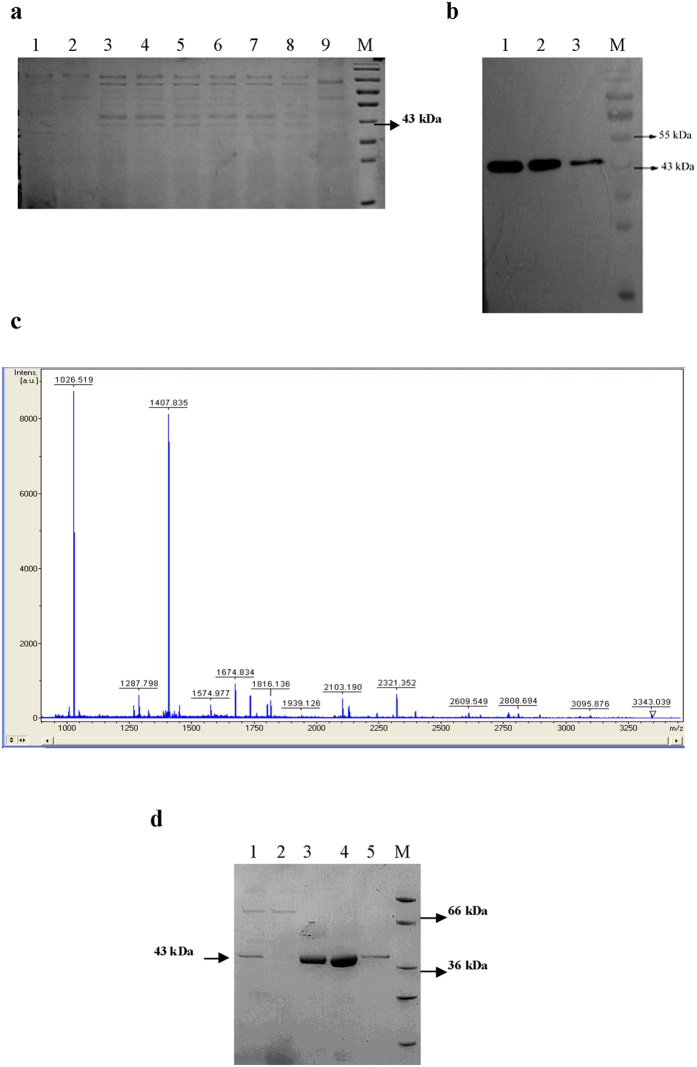
Successful expression of PprI in *Pichia pastoris*. (**a**) SDS-PAGE analysis of cultural supernatant taken from *Pichia pastoris* transformants induced with l% methanol for 3 days. Lane 1: plasmid pHBM905A transformed into *Pichia pastoris* strain GS115 (negative control). Lanes 2–9: *Pichia pastoris* strain GS115 cells NO. 1–NO. 8 transformed with pHBM905A-6 × His-*pprI*. (**b**) Western blot detection of the expression product taken from *Pichia pastoris* transformants. Lane 1: cultural supernatant of NO. 2 yeast transformant that was induced for 2 days. Lane 2: cultural supernatant of NO. 3 yeast transformant that was induced for 2 days. Lane 3: cultural supernatant of NO. 3 yeast transformant that was induced for 1 day. (**c**) PMF mapping. (**d**) The expressed and purified fusion protein PprI was analyzed using 12% SDS-PAGE followed by Coomassie Blue staining. Lane M: protein marker. Lane 1: supernatant after dialysis. Lane 2: flow through. Lane 3: elution fractions of 50 mM Tris, 300 mM NaCl, 20 mM Imidazole, pH 8.0. Lanes 4: The first elution fractions of 50 mM Tris, 300 mM NaCl, 250 mM Imidazole, pH 8.0. Lanes 5: The second elution fractions of 50 mM Tris, 300 mM NaCl, 250 mM Imidazole, pH 8.0.

Taking the preferred codons within the *Pichia pastoris* genome into account, we employed overlap-extension PCR to design and synthesize 40 pairs of DNA primers to complement a modified version of the *pprI* gene sequence from *D. radiodurans* that is optimized for expression in yeast (Supplementary Figs S1a and S2a). A polyhistidine (6 × His) tag was also introduced at the N-terminal of the *pprI* sequence. We amplified the newly synthesized *pprI* gene using PCR and connected it with the *Pichia pastoris* expression vector pHBM905A to generate the recombinant plasmid pHBM905A-*pprI* (Supplementary Fig. S1b). After the plasmid was transformed into *Pichia pastoris* strain GS115, transformant yeast cells were selected and cultured under suitable conditions. Three days later, we collected 15 μL of cultural supernatant and analyzed the secretion expression of the target protein using SDS-PAGE electrophoresis and western blotting. Compared with the negative control strains, 5 positive *Pichia pastoris* transformants exhibited bands in the 43 kDa molecular weight position specific to PprI (Fig. 1a). The western blot analysis demonstrates that the expressed protein can react specifically with the anti-6 × His tag antibody. The corresponding reaction intensity increased with an increasing methanol induction time (Fig. 1b). In addition, we carried out Peptide Mass Fingerprinting (PMF) using an Ultraflex II TOF/TOF mass spectrometer and inputted our results into the OMOSSA database of the National Center for Biotechnology Information (NCBI) (Fig. 1c and Supplementary Fig. S3). The results indicate that the expressed protein sequence is indeed consistent with that derived from *D. radiodurans* coding sequence R1 DR0167. After extending the yeast culture, we collected 1L of cultural supernatant for purification of the PprI fusion protein on Ni-NTA Spin Columns (Fig. 1d). A total of 2 mg of the target protein were ultimately extracted. We were thus able to complete an efficient expression and purification of the PprI protein (taken from the prokaryote *D. radiodurans*) in yeast.

### PprI increases the survival rate of irradiated HUVECs

**Figure 2:**
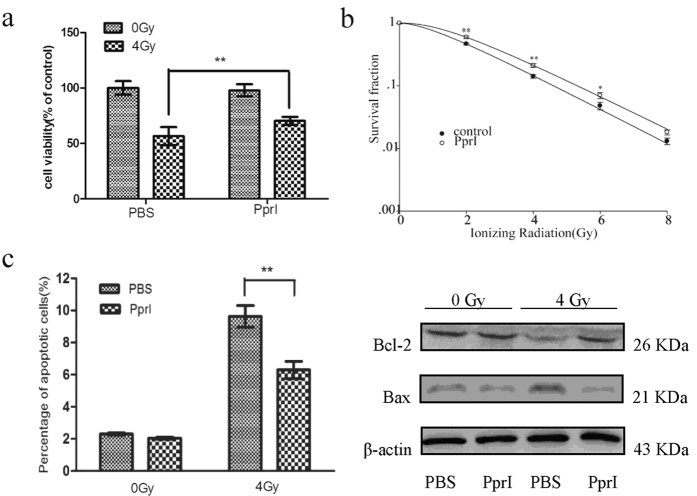
PprI protected HUVECs from radiation. (**a**) PprI-induced increase in the survival rate of HUVECs after 4 Gy γ-ray irradiation. Cell viabilities were determined with a colorimetric assay using CCK-8. Data are shown as mean ± standard deviation, and images are representative of triplicate experiments (n = 3) (*P* < 0.05). (**b**) PprI-induced increase of cell colony formation efficiency. Data are shown as mean ± standard deviation, and images are again representative of three independent experiments (n = 3) (*P* < 0.05). (**c**) Cells collected 48 h after irradiation, stained with Annexin V-FITC and propidium iodide and analyzed by flow cytometry. Data are again shown as mean ± standard deviation, and images are again representative of triplicate experiments (n = 3) (*P* < 0.05). A western blot analysis of apoptotic marker proteins in HUVECs exposed to ionizing radiation is shown at right. Cell extracts were subjected to 12% SDS-PAGE and immunoblotted with antibodies aganist Bcl-2 and Bax. β-actin was used as an internal control.

We next investigated any effects PprI might have on the radioresistance of human umbilical vein epithelial cells (HUVECs). Strikingly, cells treated with PprI prior to 4 Gy γ-ray irradiation were significantly more viable than those within a PBS-treated group (Fig. 2a). Using a colony formation assay^10^, we also counted the number of surviving HUVEC colonies over varying doses of ionizing radiation and fitted our results to a dose survival curve in accordance with the multi target-single hit model^10^,^11^. The data indicate that pretreatment with PprI results in a significant increase of HUVEC viability at both 2 Gy and 4 Gy irradiation as compared to a PBS-treated control group (Fig. 2b). The D_0_ values of the PBS and PprI treated groups were 1.5837 and 1.6597, and the corresponding D_q_ values were 0.9936 and 1.5430, respectively. In concert, these results suggest that PprI successfully improves radiation resistance in HUVECs.

We also measured the apoptosis levels in HUVECs after exposure to sham or 4 Gy γ-ray radiation. Radiation induced a dramatic increase in the percentage of apoptosed cells in PBS-treated cultures, while the addition of PprI significantly reduced this to basal apoptosis rate. The protein Bcl-2 plays an important role during the mitochondrial control of apoptosis, as overexpression of Bcl-2 can inhibit cell destruction^12^. We analyzed the expression of apoptosis-related proteins (Bcl-2 and Bax) using western blots taken 48 h after irradiation. Compared to levels in PBS-treated cells, PprI appears to reduce the expression of the pro-apoptosis protein Bax and increase the concentration of anti-apoptosis protein Bcl-2 upon cell irradiation (Fig. 2c and Supplementary Fig. S4). These findings suggest that PprI-mitigated apoptosis may be associated with modulation of the mitochondrial apoptosis pathway.

### PprI enhances antioxidant properties

**Figure 3:**
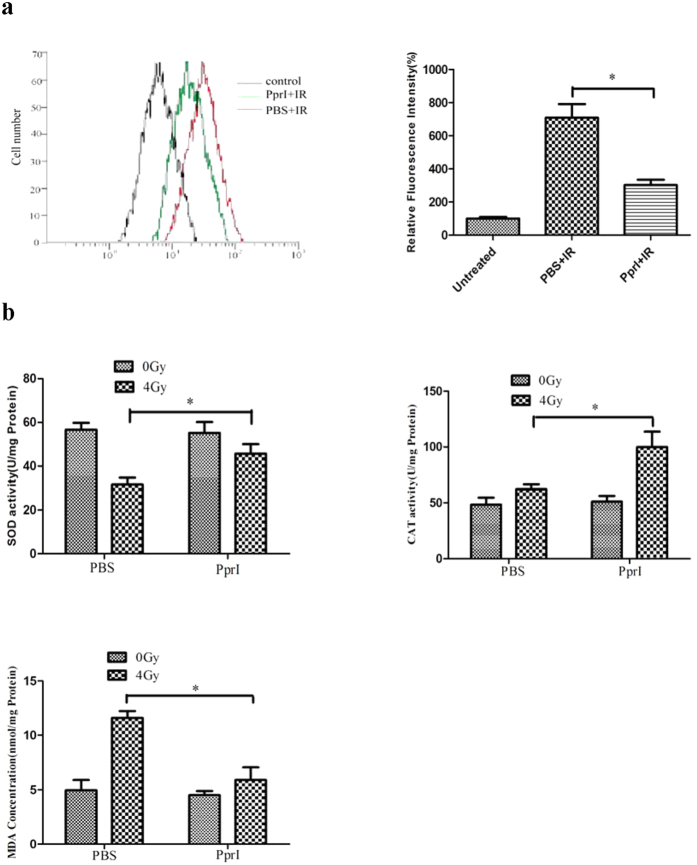
PprI enhances antioxidant properties. (**a**) Flowcytometric assessment of ROS production performed at 24 h post-irradiation. Cells were irradiated at a dose of 4 Gy; the ROS level of untreated group is set to 100%. Data are presented as mean ± standard deviation based on three independent experiments (n = 3) (*P* < 0.05). (**b**) SOD and CAT activities and MDA concentrations in HUVECs 24 h after irradiation or sham irradiation. Cells were again irradiated with a 4 Gy dose. Data are shown as mean ± standard deviation based on triplicate experiments (n = 3) (*P* < 0.05).

Oxidative stress is a crucial factor in the proliferation of DNA damage^13^. When cells are exposed to ionizing radiation or a high partial pressure of oxygen, the intracellular concentration of oxygen increases, leading to the production of reactive oxygen species (ROS) that can further damage proteins, nucleic acids, and other important elements of the cellular architecture^14^,^15^. We used the fluorescent probe DCFH-DA to detect intracellular ROS levels in irradiated HUVECs. Our results suggest that PprI serves to reduce ROS concentrations substantially (Fig. 3a). We also studied the activity of the antioxidant proteins superoxide dismutase (SOD) and catalase (CAT) within irradiated HUVECs. The enzyme SOD has the ability to degrade excess superoxide anions into less reactive species like hydrogen peroxide^16^. CAT acts to metabolize H_2_O_2_ produced by SOD or directly generated by ionizing radiation. Compared with PBS-treated cells, PprI-treated cells showed significantly increased levels of SOD and CAT activity after 4 Gy γ-ray irradiation. Additionally, we found that cells pretreated with PprI exhibited significantly decreased levels of cellular lipid peroxidation (measured through cellular malondialdehyde –MDA– concentrations), indicating a reduced degree of systemic damage (Fig. 3b).

### PprI enhances DNA repair properties

**Figure 4:**
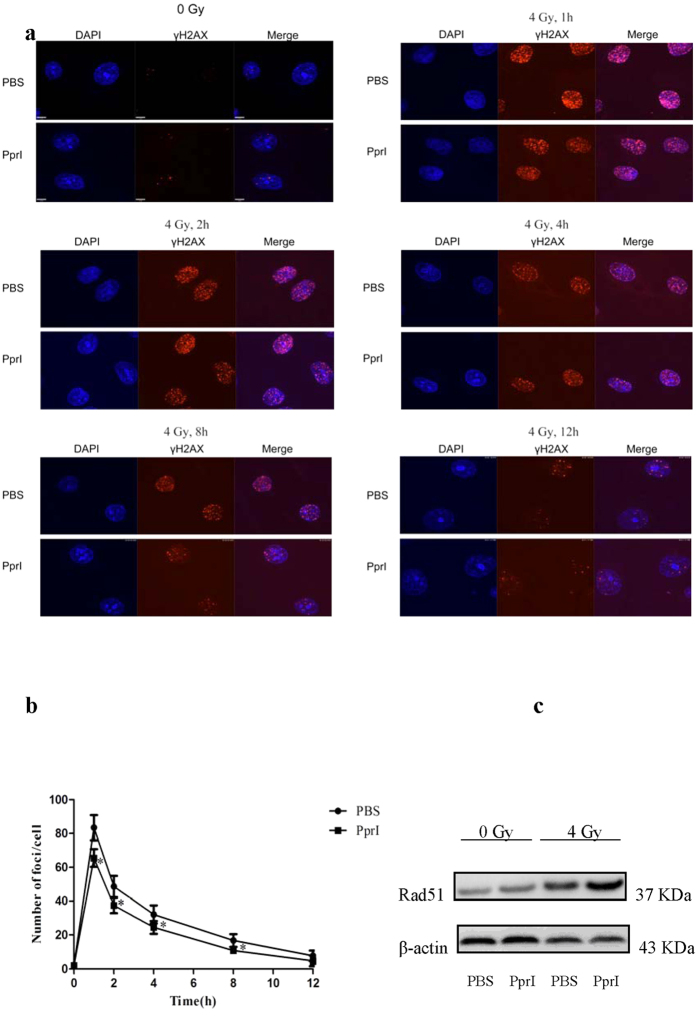
PprI enhances DNA repair properties. (**a**) Immunofluorescence assay of γH2AX foci. (**b**) Kinetics of γH2AX foci loss in different groups of cells after 4 Gy γ-ray irradiation. Data are again shown as mean ± standard deviation, and images are representative of triplicate experiments (n = 3) (*P* < 0.05). (**c**) Western blot analysis of the Rad51 protein in HUVECs exposed to ionizing radiation. β-Actin was used as an internal control.

Phosphorylation of histone H2AX on Ser139 (γH2AX) is one of the earliest markers of double-strand breakage in eukaryotes^17^. We further examined the effect of PprI treatment on radiation damage by characterizing radiation-induced γH2AX foci formation. Treated cells showed markedly reduced numbers of γH2AX foci 1, 2, 4 and 8 h after irradiation compared to the control (Fig. 4a,b). Furthermore, the protein RecA plays a very important role in homologous recombination repair in *D. Radiodurans*. Bacterial strains with defective *recA* genes are exceptionally sensitive to ionizing radiation^18^. The eukaryotic protein Rad51 protein is highly homologous to RecA protein in terms of both structure and function^19^,^20^,^21^. In a manner consistent with this observation, PprI-treated cells exhibited radically increased levels of Rad51 protein expression compared to the PBS-treated control (Fig. 4c). All of the above results suggest that the presence of PprI regulation enhances radioresistance in irradiated HUVECs.

### PprI enhances the survival rate of irradiated mice

**Figure 5:**
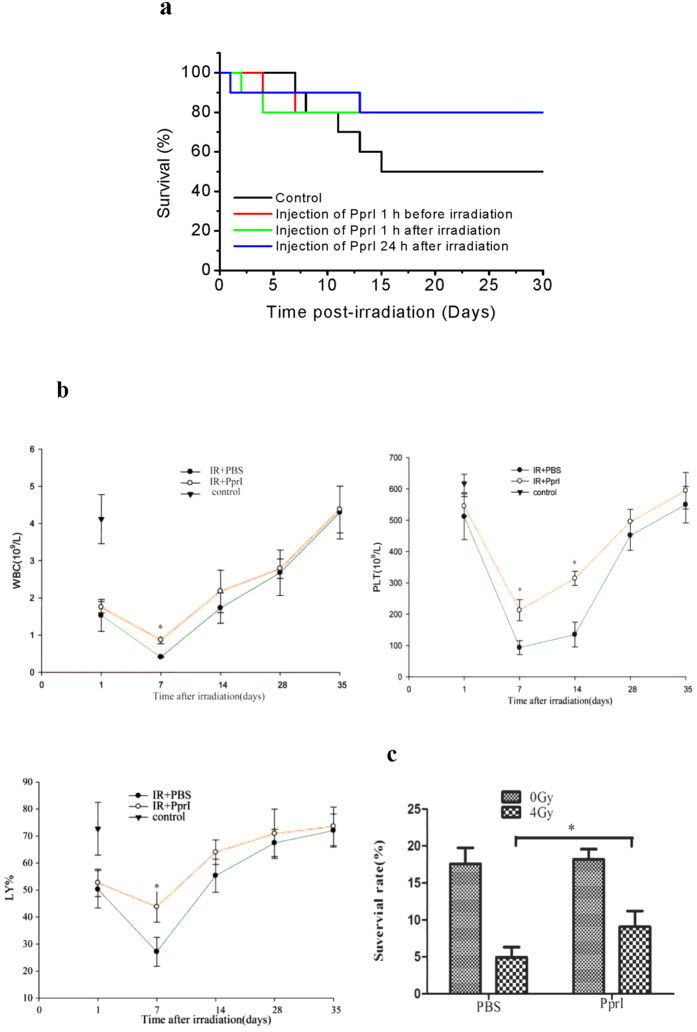
PprI-reduced mortality in irradiated mice. (**a**) The mortality of mice after 6 Gy γ-ray irradiation within 30 days (n = 10 per group). (**b**) Changes of WBC, platelet and lymphocyte percentages on days 1, 7, 14, 28 and 35 after irradiation. Data are shown as mean ± standard deviation based on triplicate experiments (n = 3) (*P* < 0.05). (**c**) Mice marrow cell clone formation rate after 0 Gy or 4 Gy irradiation. Data are again shown as mean ± standard deviation based on three independent experiments (n = 3) (*P* < 0.05).

Encouraged by our results derived from cellular radiobiology, we further investigated the efficacy of PprI treatment by studying the mortality of BALB/c mice within 30 days of 6 Gy irradiation. Within the saline control group, five of ten mice died within 15 days of irradiation, resulting in a 50% mortality rate, while only 2 mice within the PprI-treated group died within the 30 day period, reducing the mortality of irradiation to 20% (Fig. 5a).

We then examined changes in WBC, platelet, and lymphocyte counts in mice on Days 1, 7, 14, 28 and 35 after irradiation. Compared to saline control group, PprI-treated mice showed significantly improved WBC counts and lymphocyte percentages on Day 7 and increased platelet counts on Days 7 and 14 (Fig. 5b). We also observed an improved bone marrow cell clone formation rate 7 days after irradiation. The formation of bone marrow cell clones was indistinguishable between sham-irradiated mice treated with PprI or those treated with saline. However, the PprI-treated group showed substantially buffeted bone marrow cell clone formation after irradiation as compared with saline control group (Fig. 5c).

### PprI promotes repair of irradiated organs

**Figure 6:**
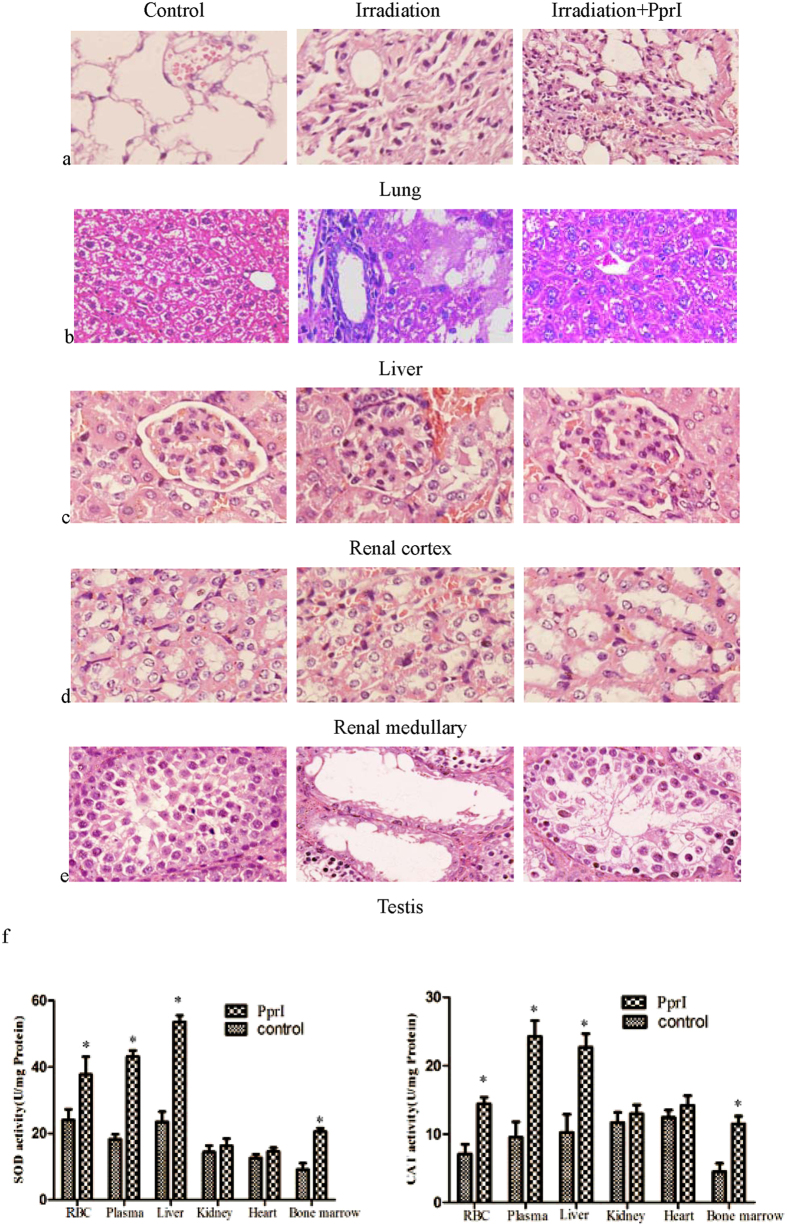
PprI promotes repair of irradiated organs. (**a**) Histopathological changes in lungs on Day 28 after 4 Gy γ-ray irradiation. (**b**) Histopathological changes in livers on Day 21 after 4 Gy irradiation. (**c**) Histopathological changes of the renal cortex on Day 28 after 4 Gy irradiation. (**d**) Histopathological changes of the renal medullary on Day 28 after 4 Gy irradiation. (**e**) Histopathological changes of the testis on Day 28 after 4 Gy irradiation. (**f**) SOD and CAT activity in different tissues 48 h after 4 Gy γ-ray irradiation. Data are shown as mean ± standard deviation based on three independent experiments (n = 3) (*P* < 0.05).

We next noted the histopathological changes that occurred in the lungs, livers, kidneys and testis of irradiated mice. The lungs of mice in the saline control group exhibited a thickening of alveolar septa by edema, an increase in fibrous tissue, and a few residual inflammatory cells on Day 28 after irradiation. Though the alveolus and its organizational structure were contorted and disorganized in the PprI treated group, only a mild inflammatory reaction in the lungs was observed. A remarkable histological recovery led to a return to normal tissue structure by Day 28 (Fig. 6a).

Marked histopathological changes in the liver occurred within the saline control group by the 21^st^ day after irradiation. Observed liver anomalies included mononuclear cell infiltration, congestion, enlargement of the veins and sinusoids, hepatocellular degeneration, severe necrotic changes, break-up of nuclei, and general disorganized tissue structure. Within the PprI-treated group, however, we observed more evidence of nuclear division and only mild increase in the number of Kupffer cells present. We saw a full return to normal liver histological structure by Day 21 in PprI-treated mice (Fig. 6b). Severe kidney damage was also observed in the saline-treated mice on Day 28 after irradiation. Glomerular capillaries exhibited vitriform degeneration and distinct tubular dilation. Hydropic degeneration was observed in the tubular epithelium alongside moderate congestion, and hemorrhaging was seen in the cortical and medulla portions of the kidney. By contrast, the kidneys of PprI-treated mice had normal structural characteristics and showed no hydropic degeneration, congestion, or hemorrhaging on Day 28 (Fig. 6c,d).

Testis, and their associated spermatogonia and spermatocytes, are highly sensitive to ionizing radiation^22^. Significant shrinkage of tubules, cytoplasmic vacuolization, and the disappearance of spermatogonia were observed on Day 28 after irradiation within the saline control group. An increase in tubular diameter was observed within the early spermatogonial population of PprI-treated mice, and the testis of mice in that group made a full recovery by Day 28 (Fig. 6e).

Lastly, we tested the functionality of the enzymes SOD and CAT in different organs and tissues of irradiated mice. Activities of SOD and CAT in RBCs, plasma, the liver, and bone marrow in PprI-treated mice were significantly higher than in the saline control group 48 h after irradiation. Activities of SOD and CAT in renal and myocardial cells, however, showed no significant difference between the two groups (Fig. 6f). These results confirm that PprI acts to enhance the function of antioxidant proteins in specific tissues in mice.

## Discussion

In recent years, studies on treatment and protection of ARI have become an important research field in the world in response to the nuclear emergency and to improve the national nuclear safety level.

*PprI* is the key gene of *D. radiodurans* in respond to DNA damage repair. Crystal structure suggests that pprI possesses three structure domains: a zinc peptidase-like domain, a helix-turn-helix motif and a GAF-like domain^23^. It was proposed that the state of pprI might change in response to DNA damage and its conformation might also be rearranged. The special constitution of the three domains in pprI indicates their synergistic effect on function and pprI is considered a generalist rather than a classic transcriptional regulator^23^,^24^. Recently, Wang *et al.* showed that the regulatory mechanism of pprI depended on its Mn^2+^-dependent protease activity toward DdrO, a transcription factor that suppresses DNA damage response (DDR) genes’ expression and relieved the repression on many DNA repair genes including *RecA, SSB* and *DdrB*. When DNA damage was fixed, pprI deactivated and DdrO reestablished the suppression of the DDR genes^25^.

Over the past 60 years, studies of radiation resistant genes and proteins of *D. radiodurans* are mainly limited in prokaryotic cells such as *D. radiodurans* itself, *Escherichia coli* and lower eukaryotes yeast systems. However, whether the expression of *D. radiodurans pprI* gene could fulfil its DNA repair function in eukaryotes and enhance the radioresistance of eukaryotes or not still remain elusive. Interestingly, Amiri *et al.* reported the expression profile of acyl-lipid Delta12-desaturase (desA) gene from Synechocystis sp. PCC6803 and its effect on cell membrane lipid composition and cold tolerance in prokaryotic (Escherichia coli) and eukaryotic (Solanum tuberosum) cells. For this purpose, a hybrid of desA and reporter gene encoding thermostable lichenase was constructed and used to transform these cells. The results showed that desaturase could enhance tolerance to cold stress in potato, and desaturase and lichenase retain their functionality in the structure of the hybrid protein where the enzymatic activity of target gene product was higher than in the case of reporter lichenase gene absence in the construction^26^. Sun *et al.* explored the effects of the human immmunodeficiency virus-1/acquired immunodeficency syndrome (HIV-1/AIDS) trans-activator of transcription (Tat) protein on human rhabdomyosarcoma cellular responses to ionizing radiation and found that HIV-1 Tat protein sensitizes cells to ionizing radiation via depressing DNA repair and dysregulating cell cycle checkpoints^27^. We wondered whether the *pprI* gene could be expressed in eukaryotic cells and whether its expression product has any effects on irradiated mammals. Using a wide variety of approaches, we have thus demonstrated that the *D. radiodurans* protein PprI effects profound radioprotective properties in human epithelial cells and mice at a systemic level. We first showed that PprI can be efficiently expressed and purified in the yeast *Pichia pastoris*, and we subsequently found that PprI has significant preventive and therapeutic effects for acute radiation injury in human and mouse cells. In both systems studied, cells treated with PprI exhibited increased survival rates and reduced rates of apoptosis after irradiation. Strikingly, activity assays suggest that PprI might impact the regulation and expression of the eukaryotic RAD51 protein (a homologue to the prokaryotic DNA-repair protein RecA). Furthermore, we observed that PprI bolsters the antioxidant response of irradiated cells, thereby mitigating secondary radiation-induced DNA damage.

In total, our results lay a theoretical and experimental foundation for the application of prokaryotic protein PprI as a radioprotective therapy in mammals, showing good prospect for the nuclear and radiation accidents, nuclear emergencies and acute radiation injury induced by tumor radiation therapy. The molecular mechanisms underlying the radiation protective effect of PprI in mammalian cells is an intriguing question and worthy of in-depth study.

## Materials and Methods

### Cell cultures

HUVECs were obtained from ScienCell Research Laboratories and cultured in an ECM medium (ScienCell) at 37 °C in a humidified atmosphere containing 5% CO_2_. Exponentially growing cells were pretreated with PBS or PprI (4 μg/mL) 6 h before irradiation and then exposed to different dosages of ionizing irradiation (according to each specific experimental procedure) using a ^60^Co teletherapy machine (GWXJ80, Nuclear Power Institute Of China) at a fixed dose rate of 0.38 Gy/min. Sham irradiated cells were assigned to the 0 Gy group. Cells treated with PBS were considered negative controls.

### Construction of pHBM-PprI expression vector and purification of PprI protein

We designed and synthesized 40 pairs of DNA primers, optimizing the CDS sequence of the *Deinococcus radiodurans pprI* gene (Gene ID: 1798483 DR_0167) according to preferred codons in *Pichia pastoris*. A 6 × His tag was cloned to the N-terminal of new synthetic *pprI* gene using the following primers: 5′-GTCACATCATCACCACCATCATGTTCCATCTGCTAACGTTTCTCC AC-3′(sense) and 5′-GGCCATTATTGGGCAGCATCTTGTGGTTCA-3′(antisense). The gene was then subcloned into the *Cop* I and *Not* I sites of pHBM905A vector (a gift from Professor Ma Lixin of Hubei University) to create the plasmid pHBM-6 × His-*pprI*. pHBM-6 × His-*pprI* was linearized by digestion with *Sal* I and transformed into *Pichia pastoris* strain GS115 using electroporation, as recommended (Invitrogen, 2008) for amplification and DNA sequence analysis. GS115 cells containing the expression plasmid were grown at 28 °C in BMGY culture for 48 h and then were induced with 1% methanol at 28.5 °C in the same BMMY culture. Three days later, 15 μL of cultural supernatant were collected to detect the secretion expression of the target protein using SDS-PAGE electrophoresis, western blotting, and MALDI-TOF mass spectrometry. His-tagged PprI protein was purified with Ni-NTA agarose (Thermo), washed with buffer containing 25 mM imidazole, and eluted with buffer containing 20 mM or 250 mM imidazole. Protein concentrations in the purified fractions were determined using a BCA assay kit (Sigma, St. Louis, MO, USA).

### Cell proliferation assay and Clonogenic survival assay

Cells were seeded in 96-well plates at 5000 cells/well and treated with PBS or PprI (4 μg/mL) 6 h before 4 Gy irradiation. 24 h later, CCK-8 solution (Dojindo Moecular Technologies Inc., Kumamoto, Japan) was added to the cells and the plates were placed in a CO_2_ incubator for an additional 2 h according to the manufacturer’s instructions. The optical density (OD) was then measured at 450 nm. Each group was set up in triplicate and cells were plated into 60 mm cell culture dishes. Either PBS or PprI was added to culture medium 6 h before irradiation, as prescribed for each experimental group. Fourteen days after irradiation, the cells were fixed and stained with Giemsa. Colonies consisting of more than 50 cells were counted as single colonies. Furthermore, the cell survival fraction was counted out and the cell survival curve was drafted by the standard model, S = 1 − (1 − e^−D/D0^)^N^ (S, cell survival fraction; D, radiation dose; e, the bottom of the natural logarithm; D_0_, the mean death dose; N, extrapolate number)^28^.

### Cell apoptosis assay

Cells were plated in triplicate into six-well plates and treated with PBS or PprI (4 μg/mL) 6 h before 4 Gy irradiation. After 48 h, cells were stained with the fluorescein FITC-conjugated dye Annexin V and PI (KeyGen, Nanjing, China) and then analyzed by flowcytometry (Beckman-Coulter, Brea, CA, USA).

### ROS generation assay

ROS concentrations in HUVECs were determined using a 2′,7′-dichlorofluorescein diacetate (DCF-DA) system (Invitrogen). After treatment, cells were washed with phosphate buffer (pH 7.4) and incubated in DCF-DA (10 μM) for 30 min. The level of DCF fluorescence was analyzed by flowcytometry (Beckman-Coulter, Brea, CA, USA).

### SOD and CAT enzyme assay and MDA concentration measurement

Cells were placed in triplicate into six-well plates and treated with either PBS or PprI (4 μg/mL) 6 h before 4 Gy irradiation. Tweenty-four hours later, SOD activity, CAT activity and MDA concentrations were determined using a T-SOD assay kit, CAT assay kit, and MDA assay kit, respectively (Nanjing Jiancheng Bioengineering Institute, Nanjing, China).

### Immunofluorescence assay

Cells were plated on glass coverslips in six-well plates for 12 h and subsequently treated with PBS or PprI (4 μg/mL) 6 h before 4 Gy irradiation. Cells were then fixed with 3.7% paraformaldehyde, permeabilized with 1% Triton X-100 (Sigma, St. Louis, MO, USA) for 15 min at 4 °C and then blocked with 5% BSA (Solarbio, Beijing, China) diluted in PBS. Anti-γH2AX antibodies (1:200, Epitomics, Burlingame, CA, USA) were incubated for 12 h at 4 °C. TRITC-conjugated anti-rabbit secondary antibodies were then incubated for 1 h at room temperature. The cells were counterstained with 4′-6-dia-midino-2-phenylindole (DAPI, Invitrogen, Carlsbad, CA, USA) to visualize the nuclei and were observed using a confocal laser scanning microscope (UltraView VoX, USA)^29^.

### Western blot

We used the following antibodies in western blot analyses: mouse anti-Bcl-2 (B3170, Sigma), mouse anti-Bax (B8429, Sigma), mouse anti-RAD51 (SAB1406364, Sigma), mouse anti-β-actin (A5316, Sigma), and lgG-Peroxidase rabbit anti-mouse (A9044, Sigma).

### Determination of mortality in irradiated mice

BALB/c mice (male, 8 weeks, SLAC Laboratory Animal, n = 10 per group) were injected intraperitoneally with either NaCl or PprI (100 μg/kg) 1 h before irradiation, 1 h after irradiation, and 24 h after irradiation. Mice were irradiated with a Co-60 γ-radiation source at a dose of 6 Gy and dose rate of 2 Gy/min. Irradiated mice were observed for 30 days and the mortality was recorded everyday. Animals were maintained in the animal facility and given ad libitum access to food and water. All of the animal experiments were approved by the Research Ethics Committee, Soochow University, Suzhou, China. All the animal experiments were conducted in accordance with the recommendations in the Guide for the Care and Use of Laboratory Animals from Research Ethics Committee, Soochow University, Suzhou, China.

### Clonogenic survival assay of mouse bone marrow

BALB/c mice (male, 8 weeks, SLAC Laboratory Animal, n = 12 per group) were injected intraperitoneally with either NaCl or PprI (100 μg/kg) 1 h after irradiation. Mice were irradiated with a Co-60 γ-radiation source at a dose of 4 Gy and dose rate of 2 Gy/min. Sham irradiation groups were injected Nacl or PprI as above. After being raised for another 7 days, mice were sacrificed, and bone marrow cells from femurs were collected and resuspended with RPMI-1640 (HyClone) to a density of 2000 cells/ml. Then the cells were mixed with 5% agar to form a 0.3% semi-solid agar medium. Cells were cultured at 37 °C in a 5% CO_2_ atmosphere. Fourteen days later, colonies consisting of more than 50 cells were counted as single colonies^30^,^31^.

### Assessment of blood cell changes in peripheral blood

Different groups of mice were irradiated with a dose of 4 Gy as described above. Blood samples were collected in EDTA tubes on days 1, 7, 14, 28 and 35 after irradiation, respectively. Samples were analyzed by a fully-automatic blood analyzer (CELL-DYN 3700, USA).

### Pathological observation of various organs in irradiated mice

Lung, liver, kidney and testis biopsy specimens from the mice in the NaCl and PprI treated groups were fixed in formalin and embedded in paraffin. Sections were cut, placed on glass slides, and stained with Harris hematoxylin and eosin. Stained tissue sections were observed under a light microscope (Olympus). Coded slides were evaluated by a single expert pathologist to determine if any histopathological changes occured.

### Determination of SOD and CAT activity of different tissues in mice

BALB/c mice (male, 8 weeks, SLAC Laboratory Animal, n = 12 per group) were injected with either NaCl or PprI, as described above. 48 h after 4 Gy γ-ray irradiation, blood, plasma, liver, kidney, heart and bone marrow were taken to measure both SOD and CAT activities.

### Statistical analysis

Results were expressed as means ± standard deviations (SD). The data was analyzed using one-way analysis of variance on SPSS 16.0 and the group means were compared by LSD Test. P < 0.05 was considered significant.

## Additional Information

**How to cite this article**: Shi, Y. *et al.* The protein PprI provides protection against radiation injury in human and mouse cells. *Sci. Rep.* **6**, 26664; doi: 10.1038/srep26664 (2016).

## Supplementary Material

Supplementary Information
