# Supplementary material for: The protein PprI provides protection against radiation injury in human and mouse cells
Source: Sci Rep. 2016 May 25;6:26664. doi: 10.1038/srep26664 (PMC4879577; doi:10.1038/srep26664)
Supplement: Supplementary Information [file srep26664-s1.pdf]

Supplementary information

The protein PprI provides protection against radiation injury in human and mouse cells

Yi Shi[1], Wei Wu[1], Huiping Qiao[1], Ling Yue[1], Lili Ren[1], Shuyu Zhang[2], Wei Yang[3], Zhanshan Yang[1]

[1]Department of Radiation Toxicology, School of Radiological Medicine and Protection, Medical College of Soochow University, Collaborative Innovation Center of Radiation Medicine of Jiangsu Higher Education Institutions, Soochow University, Suzhou, Jiangsu 215123, China

[2]Department of Radiation Genetics, School of Radiological Medicine and Protection, Medical College of Soochow University, Collaborative Innovation Center of Radiation Medicine of Jiangsu Higher Education Institutions, Soochow University, Suzhou, Jiangsu 215123, China

[3]Department of Radiobiology, School of Radiological Medicine and Protection, Medical College of Soochow University, Collaborative Innovation Center of Radiation Medicine of Jiangsu Higher Education Institutions, Soochow University, Suzhou, Jiangsu 215123, China

Correspondence to:

Wei Yang, **e-mail:** detachedy@aliyun.com

Zhanshan Yang, **e-mail:** fd@suda.edu.cn

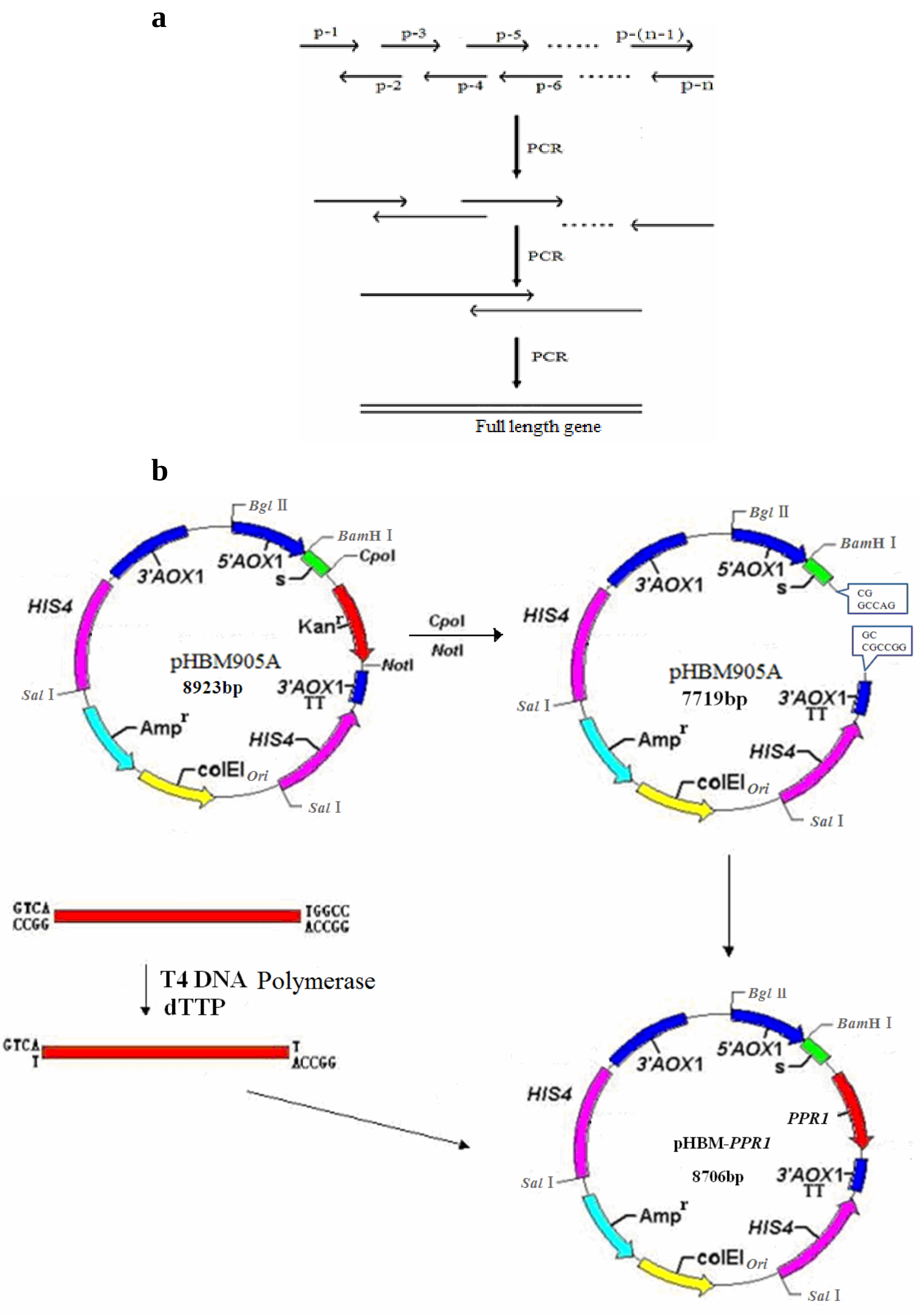

Supplementary Fig. S1. (a) Diagram of overlap-extension PCR procedure. (b) Construction scheme for the *Pichia pastoris* recombinant plasmid, integrating the modified *pprI* gene.

a

```
DR-1  GTCAGTTCCATCTGCTAACGTTTCTCCACC
DR-2  CCTCTAACACCACTTGGACATGGTGGAGAAACGTTAGCAGATGGAA  46
DR-3  ATGTCCAAGTGGTGTTAGAGGCGGTGGCATGGGTCCTAAAGCTAAG  46
DR-4  TGAGGATGTGGCTTAGAAGCCTCTGCCTTAGCTTTAGGACCCATGC  46
DR-5  GCTTCTAAGCCACATCCTCAGATACCAGTAAAGTTGCCATTTGTTA  46
DR-6  AGCGGCCAAAGCATCTGGTGCAGTAACAAATGGCAACTTTACTGGT  46
DR-7  CAGATGCTTTGGCCGCTGCTAAAGCCAGAATGCGTGATTTGGCTGC  46
DR-8  TCTACCTGGCAATGCTGCTACATAAGCGGCAGCCAAATCACGCATT  46
DR-9  GCAGCATTGCCAGGTAGAGATACTCATTCTTTGATGGCTGGTGTGC  46
DR-10 AATGGCATAAACTTCAAATCAACTCCTGGCACACCAGCCATCAAAG  46
DR-11 AGTTGATTTGAAGTTTATGCCATTGGGTTGGAGGGATGGTGCATTT  46
DR-12 AATCAGAATTACGTTATGTTCTGGATCAAATGCACCATCCCTCCAA  46
DR-13 TCCAGAACATAACGTAATTCTGATTAACAGTGCAGCTAGACCTGAA  46
DR-14 TTCATGAGCCAATGTAAATCTTTGTCTTTCAGGTCTAGCTGCACTG  46
DR-15 CAAAGATTTACATTGGCTCATGAAATCGGACATGCTATTTTGTTGG  46
DR-16 ATATCGGACAACAAATCATCATCACCCAACAAAATAGCATGTCCGA  46
DR-17 GTGATGATGATTTGTTGTCCGATATTCATGATGCTTACGAAGGTGA  46
DR-18 AGTTTCAATAACTTGTTCCAATCTCTCACCTTCGTAAGCATCATGA  46
DR-19 GAGATTGGAACAAGTTATTGAAACTTTGTGTAATGTTGCCGCAGCA  46
DR-20 CTATAACTGGTTCTGGCATCAGTATGGCTGCTGCGGCAACATTACA  46
DR-21 CTGATGCCAGAACCAGTTATAGCCGAAATGTTGGAAAGATTCGGTC  46
DR-22 ATTCTGCCAATGCTCTACCAGTTGGACCGAATCTTTCCAACATTTC  46
DR-23 TGGTAGAGCATTGGCAGAATTGGCTAAGAGAGCAGAAGTTAGTGCT  46
DR-24 CAGTCAAAGCGTACAAAGCAGAGGAAGCACTAACTTCTGCTCTCTT  46
DR-25 TGCTTTGTACGCTTTGACTGAACAAACACCAGTACCTGTTATCTAC  46
DR-26 GGCTTACCTGGAGCACAAACAGCGTAGATAACAGGTACTGGTGTTT  46
DR-27 TTGTGCTCCAGGTAAGCCTCCAAGAGAACAAGCAGCTTCCGACGAA  46
DR-28 AAACCTTTTCAGTAGATGGACCGGCATCTTCGTCGGAAGCTGCTTG  46
DR-29 GGTCCATCTACTGAAAAGGTTTTAACTGTTAGGGCCTCTTCTTCCA  46
DR-30 AAGCAAGAGTGTACTTAACACCTCTAGTGGAAGAAGAGGCCCTAAC  46
DR-31 GGTGTTAAGTACACTCTTGCTTCTGGTACTCCAGTTCCAGCTGATC  46
DR-32 CGTAGCTAATGCCAAGGCTGCAGGATGATCAGCTGGAACTGGAGTA  46
DR-33 AGCCTTGGCATTAGCTACGGGTATGGAAGTCAGAGAAGAGTCTTAC  46
DR-34 TTCCTACCAGATCTAAATGGAACGTAAGACTCTTCTCTGACTTCCA  46
DR-35 GTTCCATTTAGATCTGGTAGGAAAATGAAGGCTGAAGTTGATGCAT  46
DR-36 AACTGCAACAATTCCTCTAGATGGGTATGCATCAACTTCAGCCTTC  46
DR-37 CATCTAGAGGAATTGTTGCAGTTTCTTTTGAATTTGACCCAGCCAG  46
DR-38 CTGCTCAGAATCCTTTCTACCCAATCTGGCTGGGTCAAATTCAAAA  46
DR-39 GGTAGAAAGGATTCTGAGCAGGCTGATAGAGATGAACCACAAGATG  46
DR-40 GGCCATTATTGGGCAGCATCTTGTGGTTCA
```

b

```
atgccatctg ctaacgtttc tccaccatgt ccaagtggtg ttagaggcgg tggcatgggt   60
cctaaagcta aggcagaggc ttctaagcca catcctcaga taccagtaaa gttgccattt  120
gttactgcac cagatgcttt ggccgctgct aaagccagaa tgcgtgattt ggctgccgct  180
tatgtagcag cattgccagg tagagatact cattctttga tggctggtgt gccaggagtt  240
gatttgaagt ttatgccatt gggttggagg gatggtgcat ttgatccaga acataacgta  300
attctgatta acagtgcagc tagacctgaa agacaaagat ttacattggc tcatgaaatc  360
ggacatgcta ttttgttggg tgatgatgat ttgttgtccg atattcatga tgcttacgaa  420
ggtgagagat tggaacaagt tattgaaact ttgtgtaatg ttgccgcagc agccatactg  480
atgccagaac cagttatagc cgaaatgttg gaaagattcg gtccaactgg tagagcattg  540
gcagaattgg ctaagagagc agaagttagt gcttcctctg ctttgtacgc tttgactgaa  600
caaacaccag tacctgttat ctacgctgtt tgtgctccag gtaagcctcc aagagaacaa  660
gcagcttccg acgaagatgc cggtccatct actgaaaagg ttttaactgt tagggcctct  720
tcttccacta gaggtgttaa gtacactctt gcttctggta ctccagttcc agctgatcat  780
cctgcagcct tggcattagc tacgggtatg gaagtcagag aagagtctta cgttccattt  840
agatctggta ggaaaatgaa ggctgaagtt gatgcatacc catctagagg aattgttgca  900
gtttcttttg aatttgaccc agccagattg ggtagaaagg attctgagca ggctgataga  960
gatgaaccac aagatgctgc ccaataa                                       987
```

Supplementary Fig. S2. Sequences corresponding to (a) the 40 pairs of primers used in PCR and (b) the newly synthesized *pprI* gene.

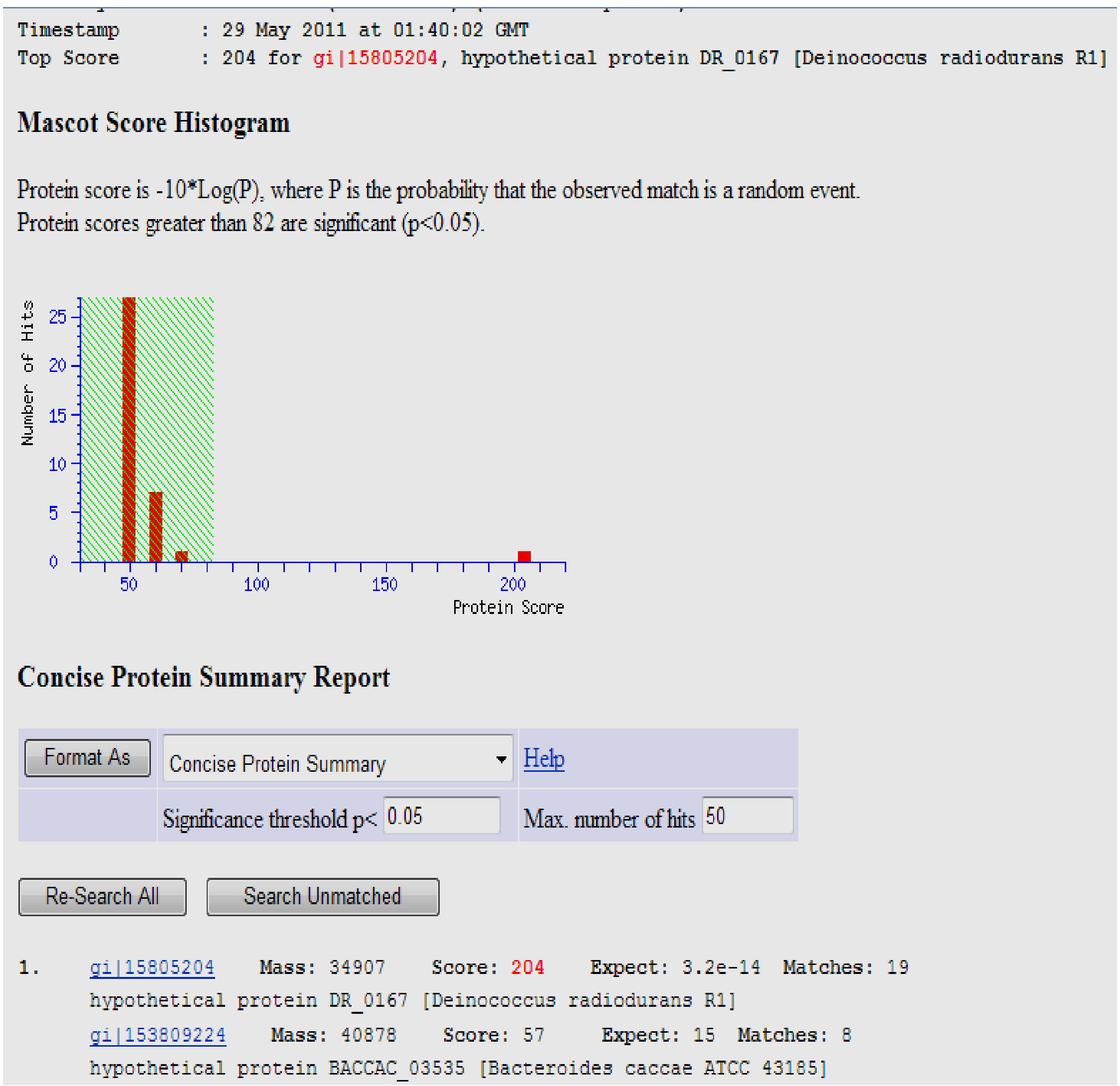

Supplementary Fig. S3. The expressed protein sequence is consistent with that derived from *D. radiodurans* coding sequence R1 DR0167.

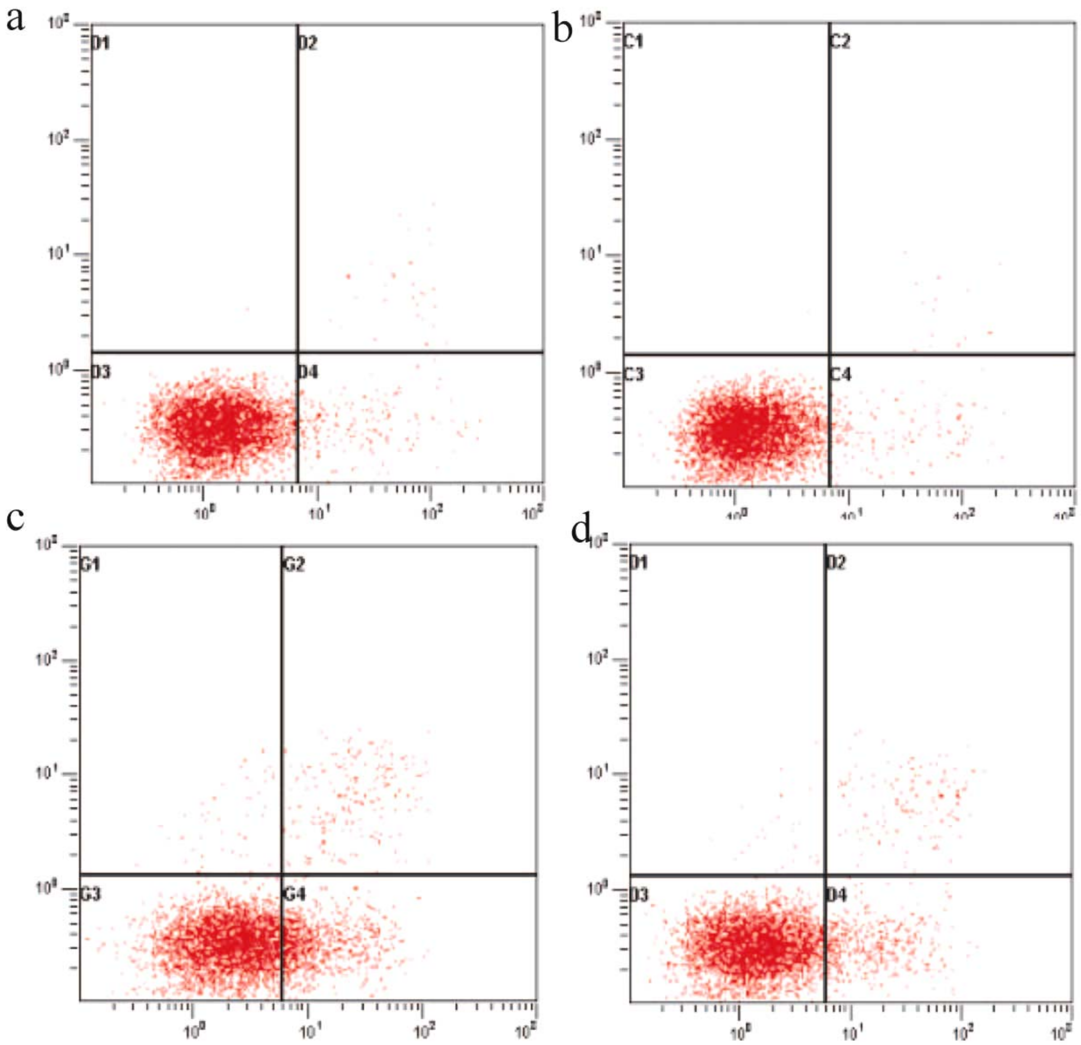

Supplementary Fig. S4. Images of apoptosis in HUVECs. Panels (a), (b), (c), and (d) represent PBS-treated (sham irradiation), PprI-treated (sham irradiation), PBS-treated (4Gy irradiation), and PprI-treated (4 Gy irradiation) cells, respectively.

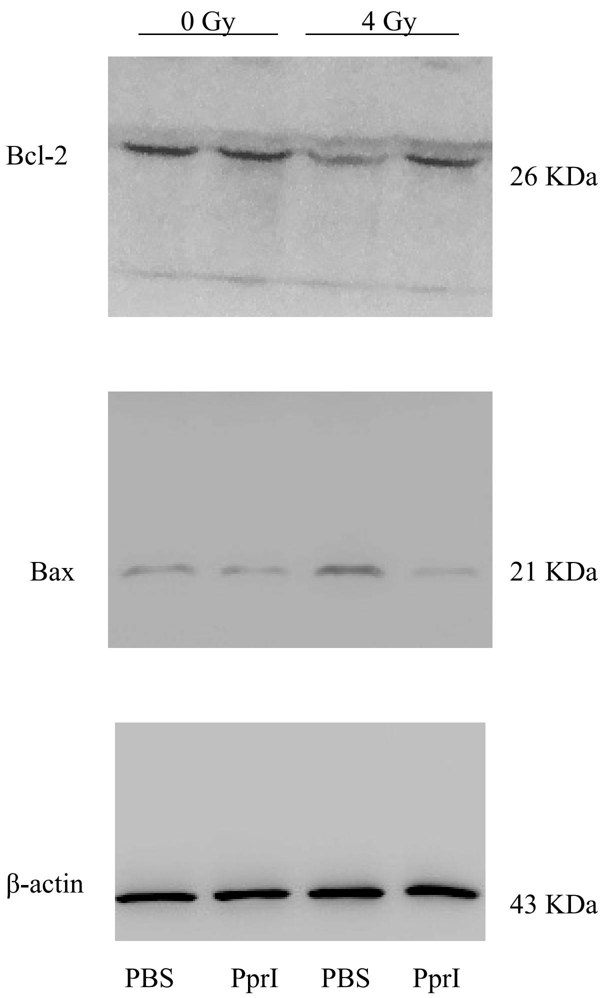

Supplementary Fig. S5. Western blot analysis of apoptotic marker proteins in HUVECs exposed to ionizing radiation (uncropped blots)

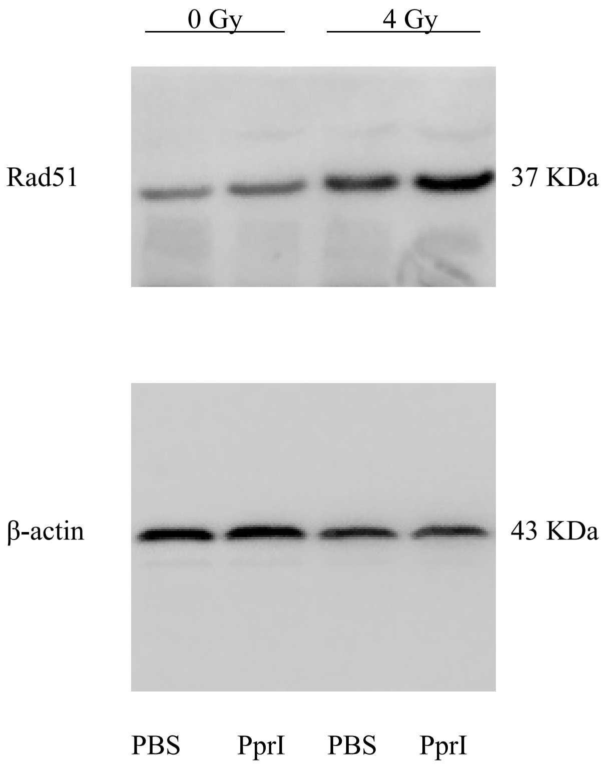

Supplementary Fig. S6. Western blot analysis of the Rad51 protein in HUVECs exposed to ionizing radiation (uncropped blots)
